# Sustainable Reinforcement for Rubbers—Potential Application of Recycled Carbon Fibers

**DOI:** 10.1021/acsomega.5c05493

**Published:** 2025-12-08

**Authors:** Péter Tamás-Bényei, Péter Sántha

**Affiliations:** † Department of Polymer Engineering, Faculty of Mechanical Engineering, Budapest University of Technology and Economics, Műegyetem rkp. 3, H-1111 Budapest, Hungary; ‡ HUN-REN-BME Research Group for Composite Science and Technology, Budapest University of Technology and Economics, Műegyetem rkp. 3, H-1111 Budapest, Hungary; § MTA-BME Lendület Sustainable Polymers Research Group, Műegyetem rkp. 3, H-1111 Budapest, Hungary

## Abstract

This study shows the utilization of recycled carbon fibers (rCF) in nitrile butadiene rubber (NBR) to produce sustainable, high-performance elastomer-based mixtures. Recycled carbon fibers, sourced from composite waste, were incorporated into the NBR matrix with different concentrations using an internal mixer for compounding and hot pressing for vulcanization. Tensile, hardness, tear and abrasion tests, and a scanning electron microscopy study were performed to show the effects of rCF. The results indicate that moderate fiber contents significantly enhance the stiffness and tensile strength of NBR without compromising its inherent elasticity. Twenty phr recycled carbon fiber increased tensile strength by 15% but decreased strain by 16% and almost doubled stiffness compared to the reference. The addition of carbon fibers caused an increase in hardness proportionally with the amount of reinforcement. 50 phr rCF increased Shore A hardness by 30%. When rCF was added, abrasion resistance increased significantly; 10 phr carbon fiber halved the amount of abraded material. Microscopic examinations confirmed the significance of fiber dispersion and adequate bonding at the matrix–fiber interface for optimal load transfer. The possibility of foaming was analyzed, and the hypothesis was proved. The results demonstrate the viability of recycled carbon fibers as a reinforcement in NBR, which also highlight the environmental and economic benefits associated with recycling composite materials in the rubber industry.

## Graphical abstract

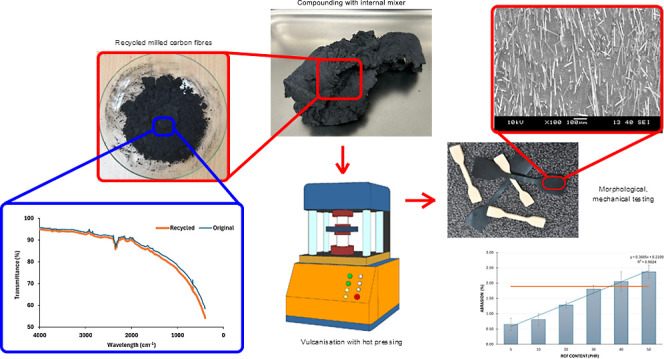

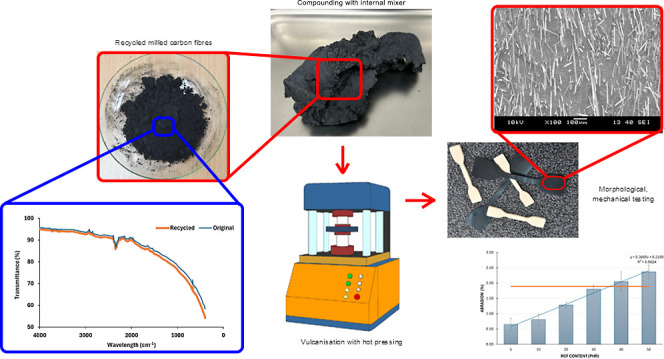

## 1 Introduction

Natural and synthetic rubbers are pivotal in various industrial applications due to their unique elastic properties and resilience. The designed characteristics of rubber products can be achieved by creating a rubber compound with many components.^1,2^ Each component is responsible for a different function,^3^ but in almost all mixtures, there are reinforcing materials. Nitrile butadiene rubber (NBR) is a synthetic copolymer known for its excellent resistance to oils, fuels, and moderate heat, making it suitable for industrial applications like seals and hoses. Unlike natural rubber (NR), which offers superior elasticity and tensile strength, NBR provides better chemical and thermal stability. The main distinction lies in NBR’s enhanced performance in oily and fuel-rich environments, while NR excels in flexibility and mechanical resilience under nonchemical exposure. The most often used reinforcements in the rubber industry are carbon black (CB) and silica. The distribution of global demand for CB is shown in Figure 1.^4^

**Figure 1:**
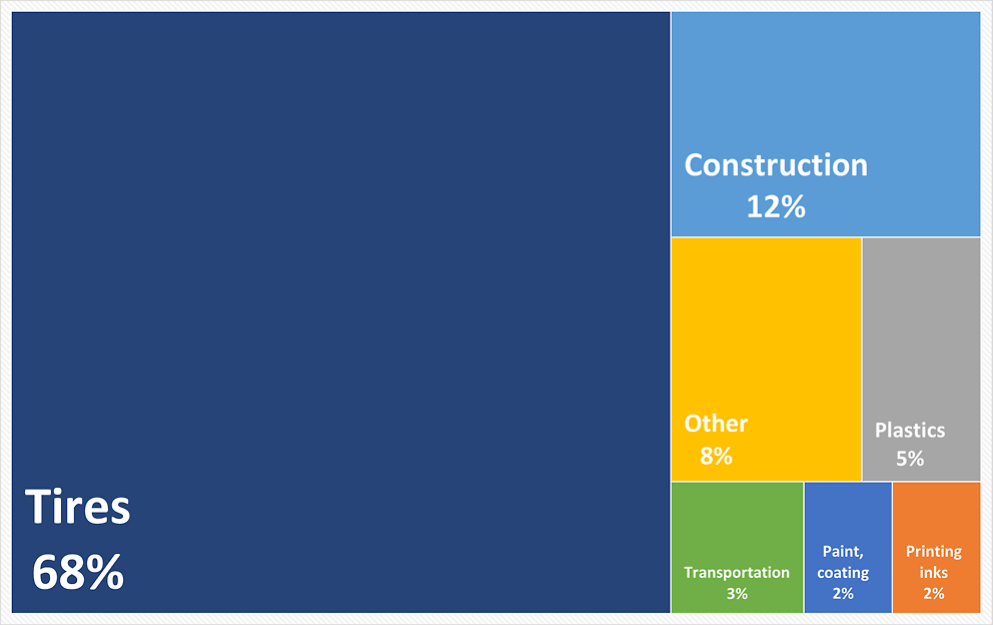
Global demand for carbon black.^4^ Reprinted in part with permission from Ceresana Market Research: Carbon Black Market Report, 2022. Copyright 2022 Ceresana e. K.-Oliver Kutsch.

Most carbon black (CB) originates from Asia and the Pacific region. Historically, a significant portion of Europe’s CB supply has been imported from areas with extensive fossil fuel industries, accounting for approximately 54% of total imports. The CB industry faces a shortage driven by complex logistical challenges and disruptions in global trade networks. These supply chain issues have resulted in delivery delays and inconsistencies, particularly as CB is increasingly routed through indirect channels such as India and China. This has occasionally led to quality deviations, which pose significant problems for the highly standardized European rubber industry. Many CB producers actively seek alternative materials and sourcing strategies in response to these persistent supply chain uncertainties and quality concerns.

In the rubber industry, replacing CB is challenging, due to its beneficial influence on the morphological and mechanical properties of rubbers.^5^ Increasing CB content enhances the tensile strength, hardness, modulus of elasticity, and tear resistance of acrylonitrile butadiene rubber (NBR). The addition of CB increases the viscosity and specific gravity of the rubber, while decreasing scorch time and cure time, indicating a faster vulcanization process.^6^ Increasing CB content enhances the creep resistance of styrene–butadiene rubber (SBR) and NBR rubber, likely due to CB agglomerates restricting the movement of rubber matrix chains. Vulcanizates with higher CB content exhibit reduced initial relaxation speeds and modulus ratios, suggesting slower stress relaxation responses.

Additionally, higher CB levels contribute to greater long-term dimensional stability under constant load, showcasing the influence of filler content on the mechanical behavior of rubber compounds.^7^ The addition of CB in SBR and NBR rubber compounds reduces swelling while increasing the residual deformation and thermal resistance.^8,9^ Increasing carbon black content results in higher electrical conductivity beyond a critical filler content (percolation threshold) due to the formation of conductive paths. There is a complex relationship between CB content, mixture viscosity, and mechanical properties of the compounds.^10–14^

The CB in rubber compounds can be best replaced completely or partly with carbon-based materials. The application of carbon fibers in rubbers is not an entirely new area. Liu et al.^15^ embedded high-modulus carbon fibers in a low-modulus silicone rubber matrix. This flexible–rigid interface significantly improved interfacial shear strength and mechanical properties, helped absorb stresses, and prevented crack propagation, slightly enhancing the thermal stability of the composites. He et al.^16^ studied the influence of carbon fiber on the wear properties of nitrile rubber-based compounds. Their results proved that carbon fibers can increase hardness and improve wear resistance. Ding et al.^17^ investigated vertically oriented CF reinforced silicone rubber to enhance thermal conductivity. Since the fibers were aligned vertically, the composites significantly improved heat dissipation compared to those with randomly oriented fibers. Wang et al.^18^ developed a mixture of rubber foam reinforced with carbon nanotubes and carbon fibers, improving both insulation properties and abrasion resistance. Enew et al.^19^ applied aramid fibers combined with carbon monofibers (CMF) and nano carbon black (NCB) in varying composition to reinforce EPDM. They found that hybrid composites combining aramid with either carbon fibers or nano carbon black further improved performance due to synergistic effects. Huang et al.^20^ focused on silicone rubber composites reinforced with low areal density carbon fibers to enhance ablation resistance. The addition of multiple layers of CF fabrics resulted in lower ablation rates, attributable to improved barrier properties and thermal stability. The researchers recognized that surface treatment is also a key issue for CF reinforced elastomers.^21^ The modification promoted stronger chemical bonding at the interface, evidenced by increased mechanical anchoring and stress transfer. Enhanced interfacial adhesion resulted in higher pullout forces in mechanical testing. Chen et al.^22^ analyzed the importance of the silane coupling agents for improving the thermal and mechanical properties of carbon fiber/natural rubber compounds. Their results showed that the silane coupling agents modified CF significantly improved tensile properties. They found that the enhanced thermal and mechanical properties of CF/NR can be attributed to improved interfacial interactions, which restrict the filler’s movement relative to the matrix and effectively lower thermal resistance at the interface.

As the literature review shows, it is not likely that CF reinforced rubbers will be widely used, due to the high price of carbon fibers and their large environmental footprint. Recently, environmental considerations have received more importance than financial aspects. However, recycled CFs provide an excellent opportunity to replace virgin CFs completely, and the CB partly or completely. Original carbon fibers are not competitive alternatives to carbon blacks due to their high price. However, reclaimed carbon fibers offer an economical replacement, as their price is a fraction of that of the original carbon fibers. Table 1 summarizes the main properties of carbon-based rubber fillers. Recycled CFs are obtained from recycling end-of-life composite products such as aircraft, automotive parts, wind turbine blades, etc. The recycling process can include different steps, such as mechanical and thermal treatments, which deteriorate mechanical properties. Thanks to the development of recovery processes, mechanical properties are degraded less than before.^23^ Applying of recycled milled CFs as reinforcements in rubber compounds presents a sustainable approach to improving material properties while addressing environmental concerns. There is some research where researchers are working on the production of sustainable fibers, such as carbon fibers from alternative sources,^24^ but either their overall ecological footprint (energy requirements for production, chemicals used) is higher, or their mechanical properties are lower than those of recycled carbon fibers, for the moment.

**Table 1:** Comparison of the Potential Carbon-Based Rubber Reinforcements^25–31^

material	particle size range	price [EUR/kg]	primary energy demand (CED) [MJ/kg]	carbon footprint [kg CO_2_-eq/kg]
carbon black (thermal decomposition sources)	∼20–100 nm	∼1–3	∼50–60 (industrial furnace processes)	∼2.3–3.4
carbon black (incomplete combustion sources)	∼20–200 nm (lamp/soot types)	similar to thermal (±20%)	similar or slightly lower efficiency	∼2.3–3.4
virgin carbon fiber (PAN-based)	filament diameter ∼5–7 μm	∼30–35	∼747 (European average)	∼25–34
recycled carbon fiber (via pyrolysis)	∼5–7 μm diameter, shorter length	∼10–20 (market price 17)	∼38–60 (pyrolysis process)	∼2.1 (with carbon capture)
MWCNT (multiwalled CNT)	diameter ∼10–50 nm, length μm-scale	∼200–400 (market estimate)	hundreds to thousands (CVD energy-intensive)	estimated ∼50–100 (high energy synthesis)

According to a comprehensive literature review and our previous results, recycled milled carbon fibers (rCF) could act as an effective reinforcement in NBR rubber compounds, improving mechanical properties such as hardness, stiffness, and tear resistance through enhanced stress transfer, provided optimized fiber content and dispersion, to avoid agglomeration and associated performance degradation. This paper investigated the effects of incorporating varying concentrations of recycled milled carbon fibers on the mechanical and morphological properties of NBR rubber mixtures.

NBR was selected for this study due to its widespread industrial relevance, particularly in applications requiring enhanced oil resistance, thermal stability, and mechanical strength. As one of the most commonly used synthetic rubbers in the automotive and aerospace industries, NBR provides an ideal matrix to evaluate the reinforcing effects of recycled carbon fibers (rCF). Its compatibility with carbon-based fillers and well-documented structure–property relationships make it a suitable benchmark for assessing sustainable reinforcement strategies. The application of the best performing mixture as a foam was also investigated.

## 2 Experimental Section

### 2.1 Materials

The PERBUNAN 3445 F acrylonitrile butadiene rubber (NBR) copolymer by Arlanxeo (Mooney viscosity: 45 MU, acrylonitrile content: 34.0 wt %; density: 0.97 g/cm^3^) and Zoltek recycled milled carbon fibers (PAN based; minimum carbon content: 92%; density: 1.81 g/cm^3^) were used. The ingredients were summarized in Table 2. The compounds were created as a potential mixture for application in the automotive industry.

**Table 2:** Materials Used for Rubber Mixtures

material	manufacturer	trademark	function
nitrile-butadiene rubber (NBR)	ARLANXEO (Geleen, Netherlands)	Perbunan 3445	elastomer
poly(ethylene glycol) (PEG)	Merck KGaA (Darmstadt, Germany)	EMPROVE PEG4000	activator
recycled milled carbon fiber (mCF)	Zoltek (Nyergesujfalu, Hungary)	Type 45	reinforcement
stearic acid (STA)	Oleon (Ertvelde, Belgium)	Radiacid 0154	accelerator activator
zinc oxide (ZnO)	Werco Metal (Zlatna, Romania)		activator
sulfur (Sul)	Ningbo Actmix Polymer (Ningbo, Zhejiang, China)	ACTMIX S-80	curing agent
dibenzothiazyl disulfide (MBTS)	Lanxess (Mannheim, Germany)	Vulkacit DM/MG-C	accelerator
azodicarbonamide (ADCA)	Avient (Berea, USA)		foaming agent

### 2.2 Test Methods

Based on the ISO 22314 standard, the size distribution of recycled milled carbon fibers was analyzed by optical microscopy in 300 single fibers. A Keyence VHX-5000 (Keyence International (Belgium) NV/SA, Mechelen, Belgium) digital microscope was used with built-in fiber length analysis software.

The spectroscopical characters of the recycled carbon fibers were analyzed with a Bruker Tensor II Fourier (FTIR) spectrometer (Bruker AXS SE, Rosenheim, Germany) and compared to those of the original carbon fibers.

A MonTech D-RPA 3000 (MonTech, Buchen, Germany) dynamic rubber process analyzer was used to analyze vulcanization characteristics. The vulcanization was characterized at 170 °C (1.67 Hz and 1° amplitude) for 20 min, and the main parameters of the vulcanization process were calculated. Scorch time (*t*10) was determined as the time required to reach 10% cure, while *t*30 and vulcanization time (*t*90) were calculated as the times to reach 30% and 90% cure, respectively. In rubber foaming, the “*t*30 time” is considered a critical parameter for understanding and controlling the foaming process, as it marks the point at which the rubber becomes sufficiently rigid to retain the expanding foam structure.

The Shore A hardness of the produced vulcanised mixtures was determined according to the ISO 7619-1 method with a Zwick/Roell H04.3150.000 hardness tester (Zwick GmbH & Co. KG, Ulm, Germany). Indentation time was 3 s, and the load was 8.05 N. Each sample was measured at 10 different points, with a 10 mm distance between each measuring point. The average and standard deviation were calculated for each material composition.

Tensile tests were performed according to ISO 37 using Type 2 dumbbell specimens to measure tensile strength, strain, and specific modulus. Tear tests were conducted in accordance with ISO 34, Method B, using angle test pieces to evaluate the material’s resistance to crack propagation. A Zwick Z005 universal testing machine (Zwick GmbH & Co. KG, Ulm, Germany) was employed for both tests, equipped with a 5 kN load cell and operated at a crosshead speed of 500 mm/min. To assess the directional dependence of the mechanical properties, specimens were tested in two mutually perpendicular orientations.

The material compositions’ abrasion resistance was measured according to the ASTM D5963-04 standard with an MV rotary drum abrasion tester (Microvision Engineering Pvt. Ltd., Rai Sonepat, India). The weight of the round samples (3 pieces from each material) was measured before and after the abrasion test. The difference between the weights, the average, and the standard deviation were calculated. Statistical mechanical properties were analyzed using one-way ANOVA to evaluate the effect of recycled carbon fiber (rCF) content, with significance set at *p* < 0.05. ANOVA was performed on the whole data set to identify overall statistical differences among the groups. Subsequently, Tukey’s post hoc tests were conducted between each sample pair to determine significant differences between specific materials.

The samples were examined using an electron microscope to support the explanation of the trends in mechanical properties. A JEOL JSM 6380LA (Jeol Ltd., Tokyo, Japan) scanning electron microscope was used for the observations. Before microscopy, the sample surfaces were sputter-coated with gold to prevent charging. Based on the micrographs, the direction of the embedded fibers was analyzed to interpret the trends observed in the mechanical test results. The fiber directions in the prepared mixtures were calculated using the image processing module of MATLAB R2024a.

### 2.3 Preparation of Rubber Mixtures

The rubber compounds were prepared with a Brabender Lab-Station internal mixer (Brabender GmbH & Co. KG, Duisburg, Germany) with an Intermix 350SX mixing chamber (350 cm^3^ free volume). The temperature was set to 110 °C, and mixing was done in two steps. First, the batches were mixed at 15 rpm for 8 min to minimize the temperature increase. Then the speed was increased to 40 rpm for 4 min for better incorporation. Figure 2 shows the production process of mixtures and samples. Two mm thick vulcanised rubber sheets were produced from the compounds with a Teach-Line Platen Press 200E hot press (Dr. Collin GmbH, München, Germany) at 170 °C and 200 bar. Pressing time was based on the vulcanization time (*t*90) specific to each compound. Afterward, the specimens were cut from these sheets using standardized dies to characterize of mechanical properties.

**Figure 2:**
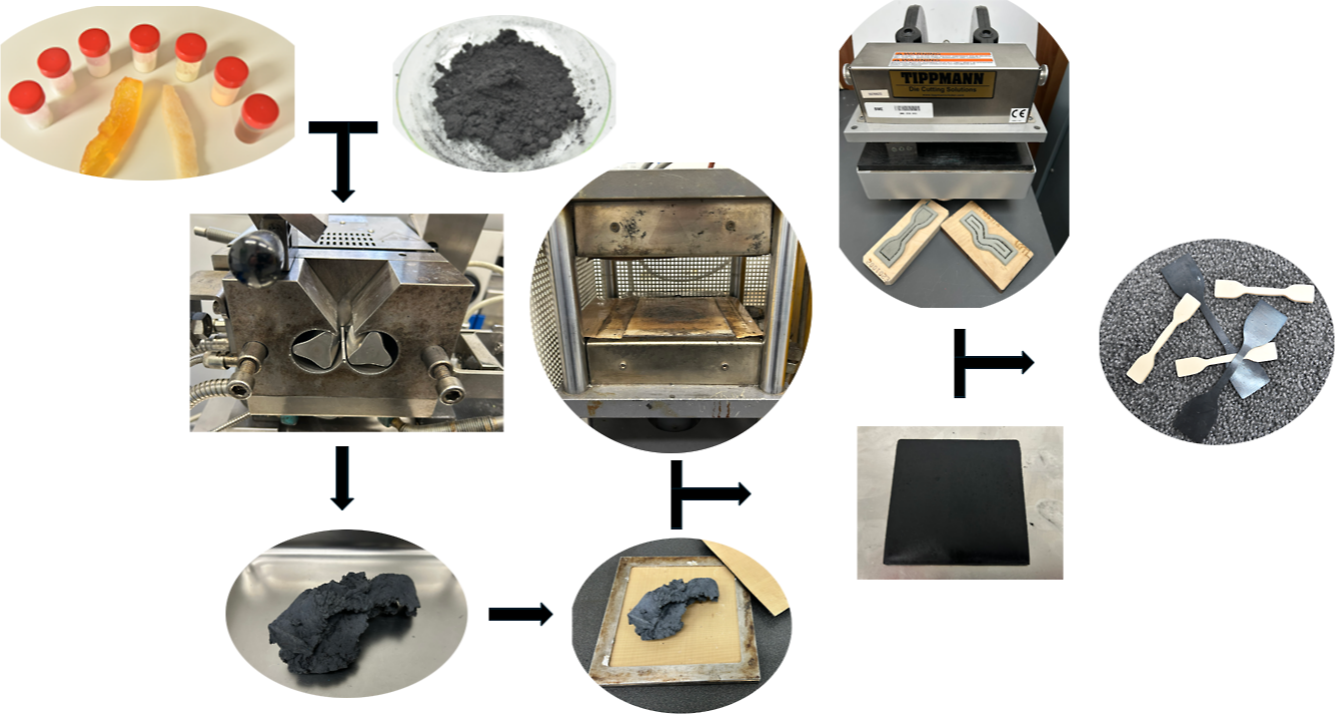
Production process of mixtures and samples.

Table 3 shows the composition of the mixtures. Based on preliminary experiments, eight recipes (including the one with the foaming agent) were formulated, and the amount of recycled milled carbon fibers varied.

**Table 3:** Recipes of the Rubber Compounds

	amount of ingredients (phr)						
material	REF	B	C	D	E	F	G
NBR	100	100	100	100	100	100	100
PEG	4	4	4	4	4	4	4
mCF	0	5	10	20	30	40	50
STA	1	1	1	1	1	1	1
ZnO	5	5	5	5	5	5	5
Sul	1.5	1.5	1.5	1.5	1.5	1.5	1.5
MBTS	2.5	2.5	2.5	2.5	2.5	2.5	2.5

## 3 Results and Discussions

### 3.1 Recycled Carbon Fibers

Figure 3 illustrates the statistical distribution of the detected fiber sizes in microns. The 60–74 μm range has the highest count, with approximately 70 fibers. The 45–59 and 75–89 μm ranges are also prevalent, indicating that medium-sized fibers are the most numerous. In contrast, fibers smaller than 44 μm are rare, and the number of fibers increases significantly up to 74 μm. Over 90 μm, the number of fibers gradually decreases, with fibers larger than 210 μm being rare. Overall, the data indicate that medium-sized fibers dominate. Based on the statistical analysis, the average fiber length is 70.05 ± 1.12 μm and the aspect ratio is about 12 ± 2.

**Figure 3:**
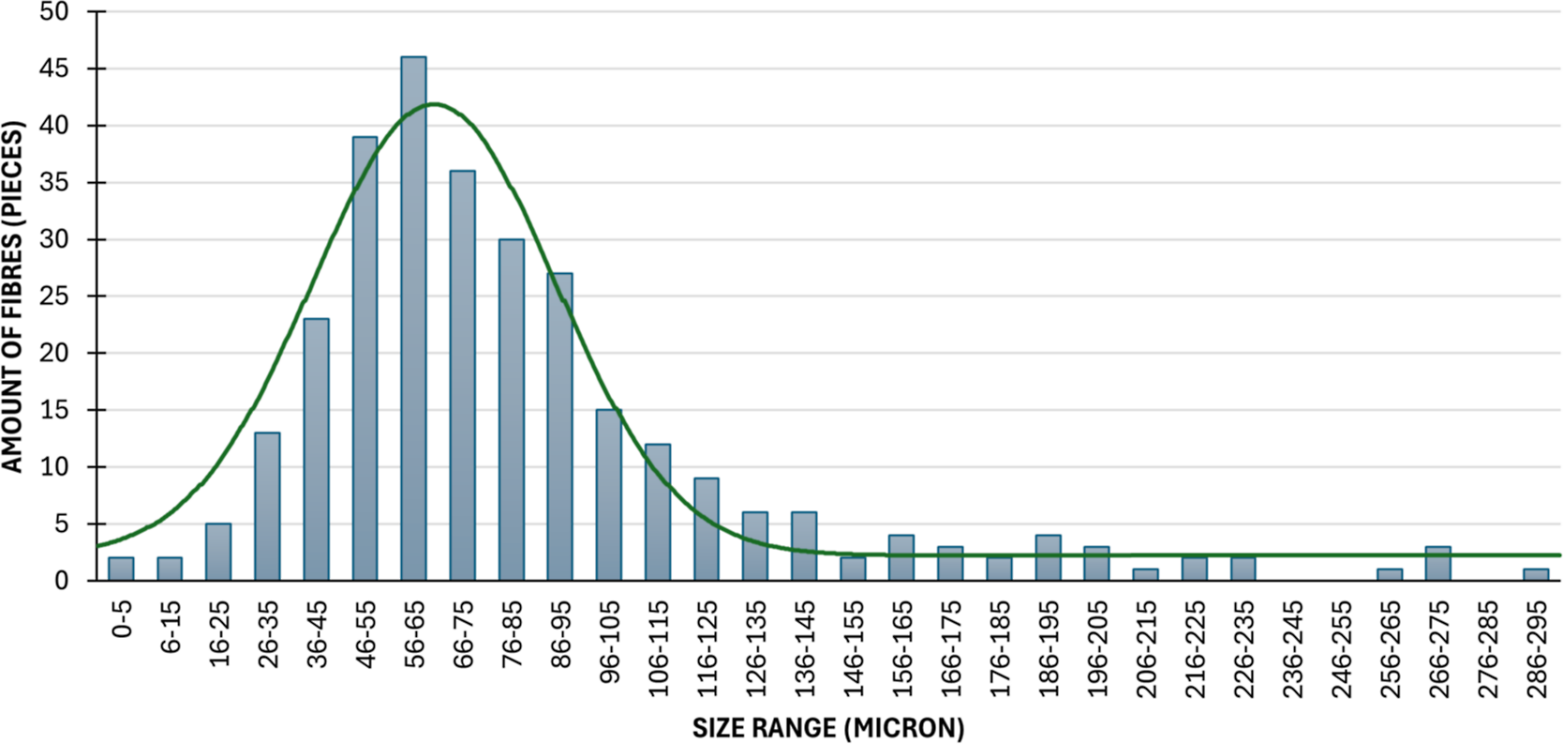
Size distribution of recycled milled fibers.

The FTIR spectra for the recycled and the original fibers (Figure 4) show a weak, broad band around 3400 cm^–1^, which suggests O–H stretching from hydroxyl groups or absorbed moisture. It was found that the characteristics of the materials do not differ significantly from each other. A small peak near 2920 cm^–1^ indicates C–H stretching, potentially from sizing agents or minor organic residues. A distinct absorption near 2350 cm^–1^ is attributed to the asymmetric stretching of atmospheric CO_2_, a common feature in FTIR measurements and unrelated to the fiber chemistry. The weak absorption around 1700–1740 cm^–1^ points to C=O stretching, often associated with carboxylic or carbonyl groups formed during processing or oxidation. The region near 1100–1300 cm^–1^ can be attributed to C–O stretching vibrations, possibly linked to residual epoxy or other surface treatments. Overall, the spectra exhibit only minor differences in peak intensities, indicating that the recycling process does not drastically alter the material’s chemical structure.

**Figure 4:**
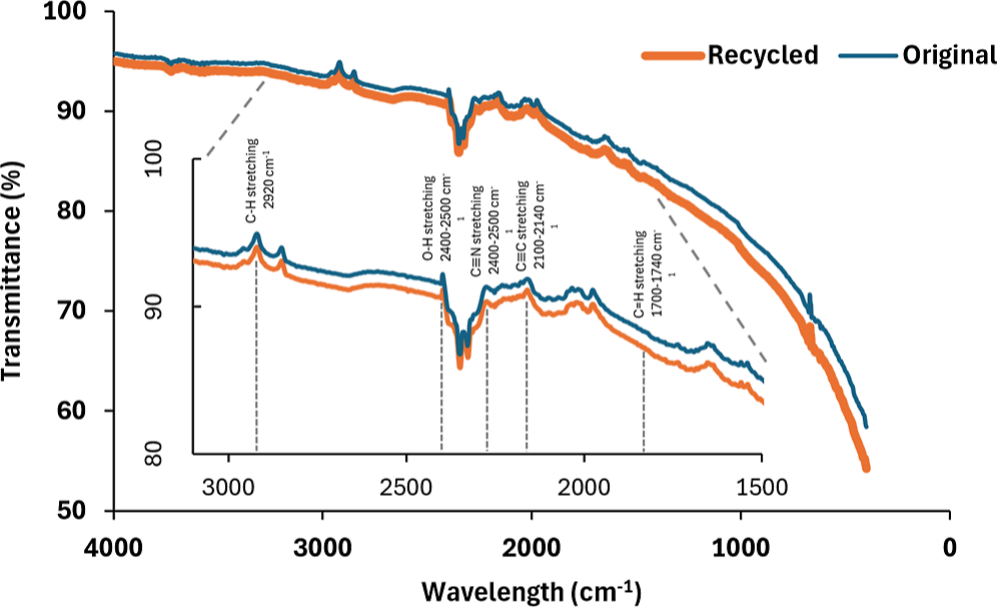
FTIR spectra of original and recycled carbon fibers.

### 3.2 Uncured Compounds

Figure 5 represents the vulcanization curves of the mixtures and summarizes the characteristic times. The graphs display the torque (Nm) as a function of time (s) for the prepared samples (REF, B, C, D, E, F, and G). All curves start at a comparably low torque and decline before a steep ascent. REF exhibits the slowest rise and attains the lowest maximum torque compared to the other samples. The maximum torque of sample G is the highest, followed closely by F and E. The differences between the samples become more pronounced in the initial 400 s, stabilizing near 1200 s. It is evident that REF consistently demonstrates an inferior torque response relative to the other samples. The graph indicates apparent differences in material or system behavior across the samples. The results indicate that increasing reinforcement causes curing time to decrease and the measured torque to increase, which causes a higher torque need for homogeneous mixing. REF is the slowest in all cases, taking 82 s for TC 10, 113 s for TC 30, and 381 s for TC 90. Material G is the fastest overall, with TC 10 at 46 s, TC 30 at 59 s, and TC 90 at 178 s. The increasing rCF content, which increased thermal conductivity and accelerated curing, can explain the results.

**Figure 5:**
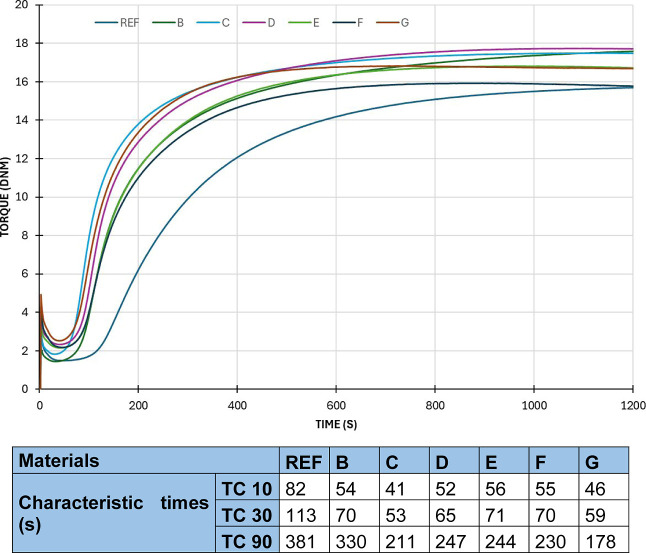
Vulcanization curves and characteristic times of the mixtures produced.

### 3.3 Vulcanized Mixtures

The results of the Shore A hardness test (Figure 6) indicate that as recycled milled carbon fiber (rCF) content increases, Shore A hardness also increases. The increase in Shore A hardness with the incorporation of rCF can be attributed to the role of rCF as a reinforcing additive, thereby enhancing material’s rigidity. Increased rCF content enhances interaction between the reinforcement and the polymer matrix, restricting polymer chain movement and increasing hardness. This phenomenon is associated with the rigidity of individuals carbon fibers. It has been found that the addition of more rCF reduces the proportion of the softer polymer, thereby shifting the material’s behavior toward that of the more rigid reinforcement. This rise in hardness indicates enhanced structural rigidity and mechanical strength resulting from higher rCF content. The addition of carbon fibers caused an increase in hardness proportionally with the amount of reinforcement. The effect of rCF can be represented with a linear trend line.

**Figure 6:**
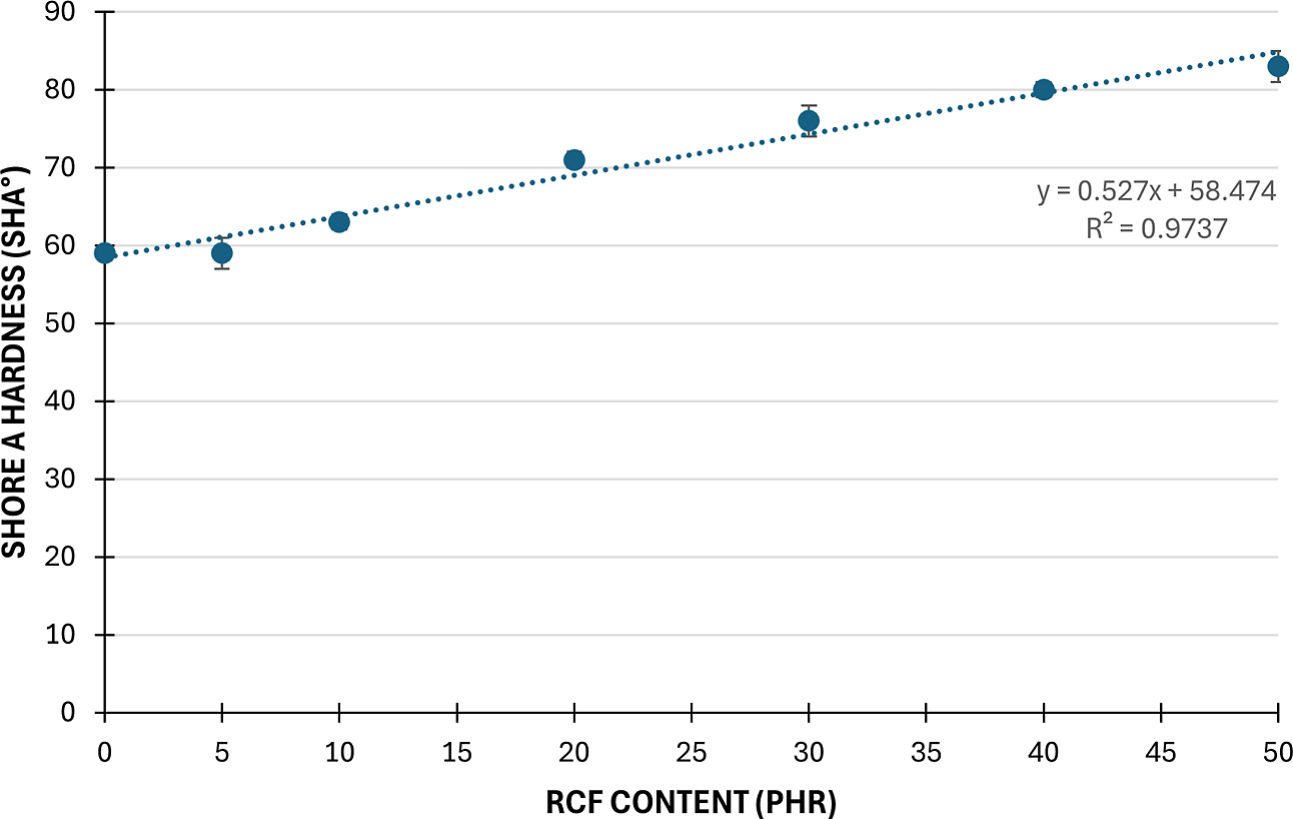
Results of Shore A hardness tests.

Table 4 shows the results of the tensile tests. The results indicated no significant difference between the two testing directions. The lowest *p*-value obtained from the ANOVA to assess directional dependency was *P* = 0.4099, well above the 0.05 significance threshold. Therefore, tensile test results were averaged across all specimens regardless of orientation. Adding recycled carbon fiber (rCF) to NBR results in a statistically significant difference in tensile strength among the material groups, as indicated by the ANOVA test (*p* = 0.028). However, this difference does not reflect a consistent trend with increasing rCF content, as most compositions’ tensile strength remains relatively constant. The highest tensile strength among the reinforced samples (5.467 MPa) was observed at 30 phr. According to the posthoc Tukey analysis, no statistically significant difference exists between the reference material and samples B, C, D, or E. Only the lowest-performing materials, F and G, which contain the highest fiber contents, are significantly weaker than the reference. This reduction in strength may be attributed to fiber agglomeration and the increased number of single fiber ends. Although the average strain at break appeared to increase slightly with higher rCF content—rising from 451% for the reference material to 500% at 40 phr—the variation remained within overlapping standard deviations. To statistically assess these observations, a one-way ANOVA was performed, yielding a *p*-value of 0.978 and an *F*-value of 0.194, both indicating no significant differences among the groups. As the *p*-value is well above the 0.05 threshold and the *F*-value falls far below the critical value (*F*_crit_ = 2.242), it can be concluded that the changes in elongation at break are not statistically significant. These findings suggest that the addition of rCF does not adversely affect the ductility of the material, and the composite maintains consistent failure strain characteristics across all investigated fiber contents. ANOVA results show that recycled carbon fiber (rCF) content significantly affects M100 (modulus at 100% elongation) (*F* = 2.75, *p* = 0.019) and M200 (*F* = 3.27, *p* = 0.007). Tukey tests reveal that M100 is significantly lower for 5 and 10 phr rCF groups compared to the reference, while higher rCF levels show no significant difference. M200 of all rCF groups have significantly reduced versus the reference, but differences among rCF groups are not significant. Overall, adding rCF lowers modulus, especially at low filler content for 100% and across all contents at 200% elongation. The results suggest that rCF behaves primarily as a filler rather than a reinforcement in this system, particularly at higher elongations. The reduction in modulus is probably due to a combination of poor fiber–matrix adhesion, inadequate dispersion at low loadings.

**Table 4:** Results of the Tensile Tests

materials	rCF content (phr)	tensile strength (MPa)	strain at break (%)	M100 (MPa)	M200 (MPa)
REF	0	6.01 ± 1.15	451 ± 105	2.46 ± 0.71	3.87 ± 1.14
B	5	5.26 ± 0.78	487 ± 141	1.84 ± 0.48	2.79 ± 0.66
C	10	5.11 ± 1.27	467 ± 163	2.01 ± 0.30	2.79 ± 0.46
D	20	5.33 ± 0.74	493 ± 105	2.37 ± 0.53	3.02 ± 0.69
E	30	5.47 ± 1.05	490 ± 89	2.53 ± 0.65	3.07 ± 0.75
F	40	4.60 ± 0.68	500 ± 97	2.29 ± 0.27	2.62 ± 0.29
G	50	4.57 ± 0.72	494 ± 72	2.44 ± 0.40	2.75 ± 0.41
ANOVA *p*-value (*p*crit ≤ 0.05)		0.028	0.977	0.019	0.007
ANOVA *F*-value (*F*crit = 2.242)		2.544	0.193	2.749	3.271

Figure 7 shows the results of the tearing test. The tear strength results show a clear trend of increasing resistance with higher recycled carbon fiber (rCF) content. The reference sample (0 phr) has the lowest tear strength at 12.32 kN/m, while the maximum is observed at 22.15 kN/m for the 50 phr (G) sample. ANOVA confirms that these differences are statistically significant (*F* = 6.27, *p* = 0.0007). The increase in tear strength is most notable between 5 phr and 10 phr, suggesting that even moderate rCF additions substantially improve the material’s ability to resist tearing. This enhancement is likely due to improved fiber–matrix interaction and energy dissipation during crack propagation. The uniform dispersion of rCF at moderate-to-high levels ensures even stress distribution, contributing to higher tear strength. The trend suggests that the reinforcement effect of rCF, combined with improved stress transfer and crack resistance, is responsible for the increased tear strength as rCF content increases. However, a high standard deviation was observed, suggesting that the recycled carbon fibers did not disperse uniformly or that the rubber did not adhere properly to the reinforcement.

**Figure 7:**
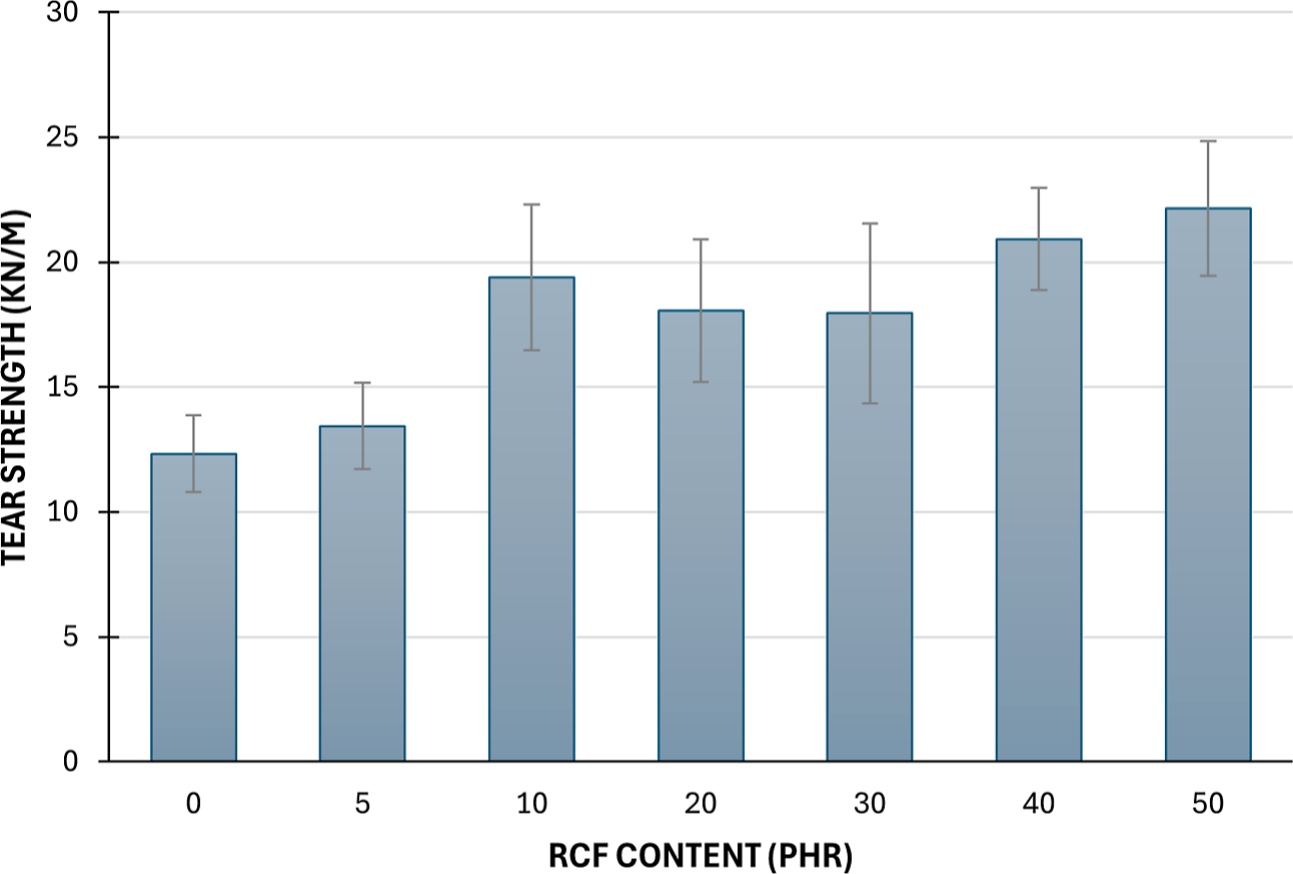
Results of the tear tests.

Figure 8 illustrates the correlation between recycled carbon fiber (rCF) content and abrasion percentage, revealing how rCF influences material wear. Abrasion resistance was significantly affected by rCF content (ANOVA: *F* = 17.17, *p* = 0.0015), with values rising from 1.89% in the reference (0 phr) to 2.37% at 50 phr (G). The Tukey post hoc test identified significant differences only between the reference and the 5 phr (B) and 10 phr (C) groups (*Q* = 6.55 and 5.70, respectively), suggesting that low rCF content notably improves wear resistance. No statistically significant differences were observed between the reference and higher rCF levels (30–50 phr), despite a clear upward trend in abrasion. While abrasion is highest without reinforcement (0 phr), it decreases at 5 phr, indicating enhanced wear performance due to fiber reinforcement. FTIR spectres show evidence for presence of polar groups (hydroxyl groups at ∼3400 cm^–1^, carbonyl groups at ∼1700 cm^–1^ and ethers/esters group at ∼1200–1000 cm^–1^) on the surface of carbon fibers. However, abrasion increases again at higher rCF levels, exceeding that of the unreinforced sample, likely due to poor fiber–matrix bonding or rCF agglomeration, which can lead to localized stress and greater surface damage. Thus, although small additions of rCF (around 5 phr) optimize abrasion resistance, excessive reinforcement may compromise it. This demonstrates that the secondary bonds between the polar rubber and the polar groups of the rCF play a significant role in enhancing tribological properties. Variability in the results—seen in the error bars—may stem from inconsistent processing or uneven fiber dispersion. In summary, while low rCF levels enhance wear resistance, higher contents lead to diminishing returns and increased material wear. At higher rCF loadings, the formation of fiber bundles and agglomerates becomes increasingly pronounced, impairs matrix penetration and reduces the effective interfacial bonding area. This agglomeration limits stress transfer efficiency and accounts for the lack of proportional improvement in tensile strength and modulus with increasing rCF content. Furthermore, the presence of such aggregates contributes to deviations from the conventional hardness–modulus relationship, as localized stiff regions enhance hardness without uniformly reinforcing the matrix. In addition, agglomerated fibers act as abrasive microparticles during wear, explaining the elevated abrasion values observed at higher rCF concentrations.

**Figure 8:**
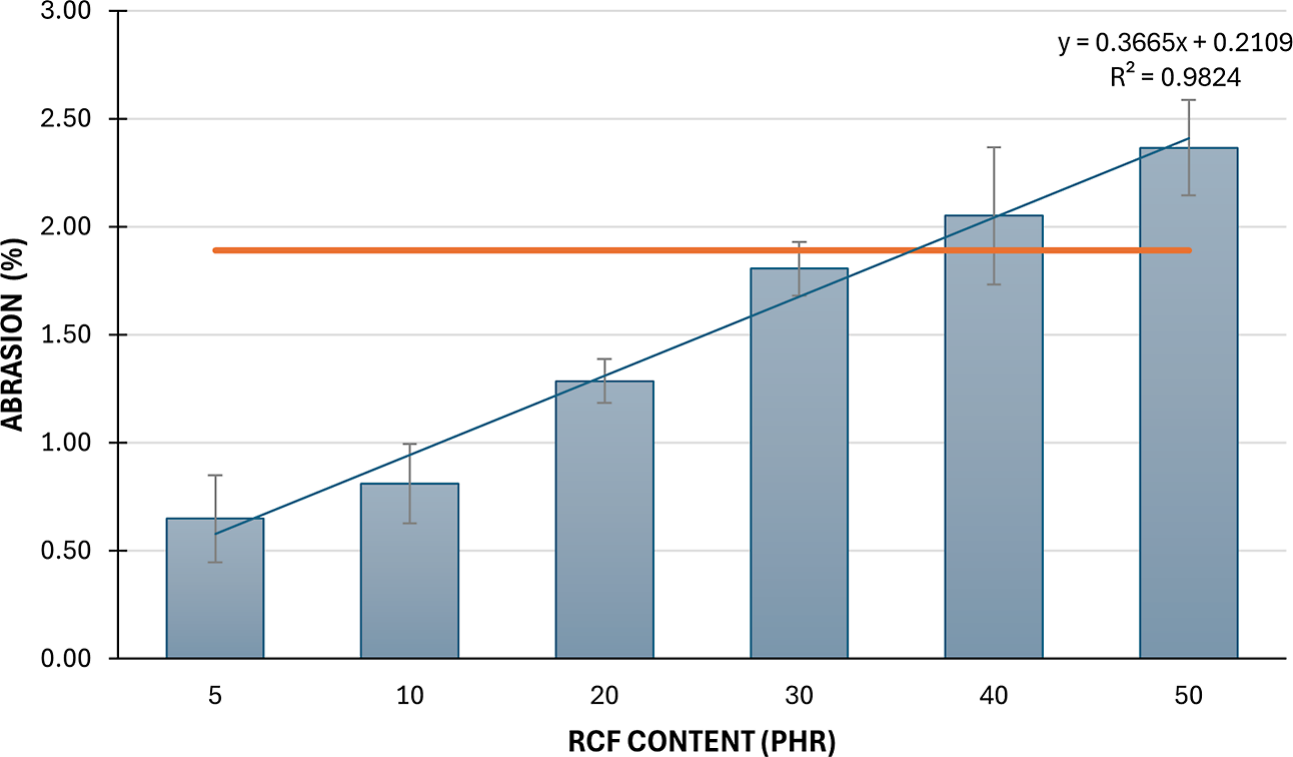
Results of abrasion tests.

The SEM images (Figure 9) A to G illustrate the distribution and interaction of recycled carbon fibers in the rubber matrix. Image A shows the samples without carbon fibers. Image B shows low rCF content with scattered fibers and substantial gaps, indicating inadequate adhesion. Image B shows a slightly enhanced fiber distribution but exhibits little reinforcement effect. Images C and D show moderate rCF content, increased fiber density, and improved alignment. rCF content is high in images E and F, with densely packed and overlapping fibers. However, these images also reveal fiber clustering and uneven distribution, which could create localized stress points and reduce the uniformity of the elastomer. Image G shows very high fiber density with a more random orientation, potentially improving isotropic properties but risking weaker directional performance. Increasing rCF content may improve overall mechanical performance, but careful dispersion is required to avoid agglomeration. Moderate rCF content (C and D) appears optimal for balancing the reinforcing effect and uniformity. High fiber content (E, F, G) has been shown to increase the risk of stress concentrations. Figure 10 shows SEM images at higher magnification, which confirm that the fibers are well embedded in the matrix material. Still, cavities can also be observed, which are the locations of fibers pulled out due to high deformation. Although the fiber–matrix adhesion can be considered adequate, it would be advisible to improve the fiber–matrix interaction through surface treatment in order to better utilize the mechanical properties of the fibers.

**Figure 9:**
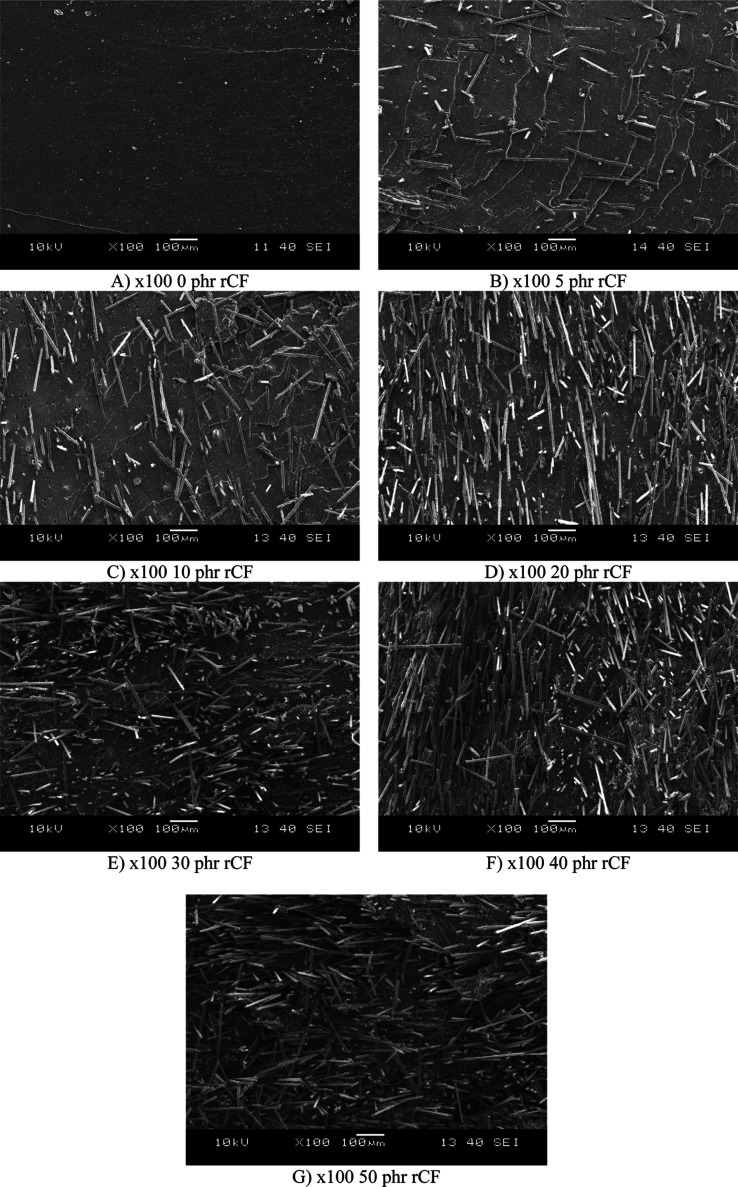
Scanning electron microscope images of the samples.

**Figure 10:**
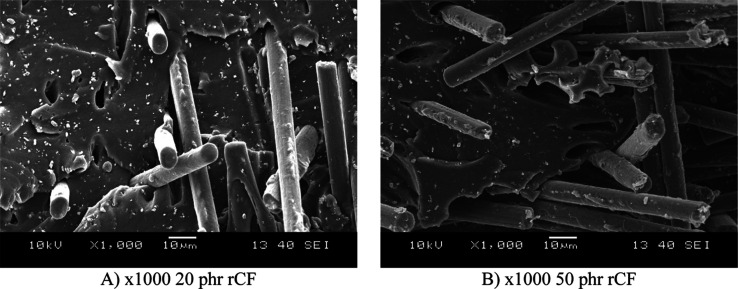
Scanning electron microscope images of the samples at higher resolution.

The radar plot (Figure 11) shows the fiber orientation distribution at different fiber contents (5, 10, 20, 30, 40, and 50 phr). Fibers exhibit more random orientation with limited alignment at low fiber contents (5 and 10 phr), indicating weaker reinforcement potential. Conversely, at 20 and 30 phr fiber content, the orientation of the fibers is balanced, with well-defined orientation peaks in the 0/180° direction, providing a beneficial combination of alignment-driven reinforcement and sufficient isotropy to enhance mechanical performance. This shows a good orientation balance at intermediate loadings, which likely contributes to the peak mechanical properties by exploiting the reinforcing potential of fiber alignment without excessive anisotropy. However, agglomeration and excessive fiber orientation reduce isotropy at higher fiber content (40 and 50 phr), potentially creating stress concentrations and weakening mechanical performance due to excessive directional behavior. It is important to note that the measured mechanical properties did not exhibit anisotropic behavior. However, SEM images reveal apparent fiber orientation differences. This discrepancy arises because SEM captures localized regions of agglomerated fibers that may be preferentially aligned in certain directions. These local orientations do not affect the bulk material, which remains isotropic in its mechanical response. Our findings highlight the necessity of optimized fiber distribution and matrix bonding to achieve uniform mechanical properties and effective reinforcement. According to the results of the SEM study, the adhesion between the rubber and fibers is a key factor in achieving better mechanical performance. Grafting with a silane-based coupling agent is the most promising among the potential methods to improve the adhesion. Further research will investigate the influence of surface treatment on mixture properties.

**Figure 11:**
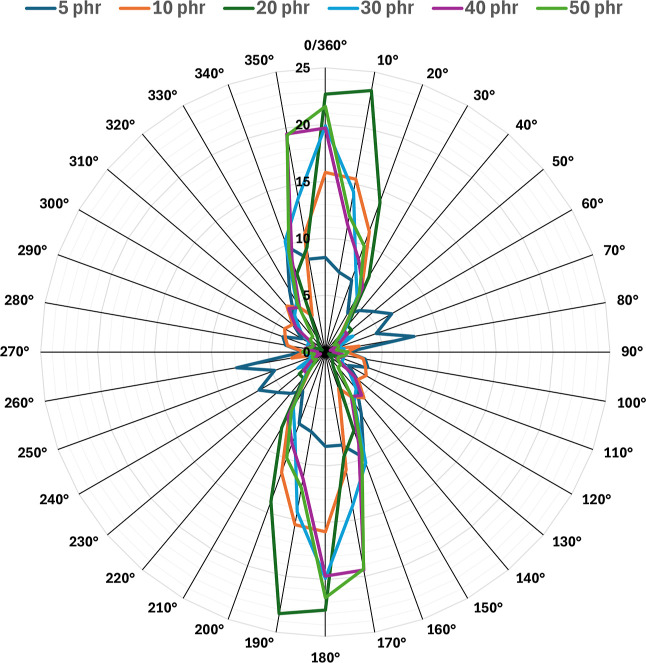
Results of fiber direction analysis.

### 3.4 Rubber Foam

Based on the results of the mechanical tests, the original mixture “D” (without a foaming agent) was selected for foaming (Table 5). A foaming agent was added to the original recipe. The mixture was produced by internal mixing, as previously described. Following compounding, the samples were foamed. A complex three-step foaming process was developed (130 °C for 3 min, followed by 165 °C for 9 min, and then 175 °C for 5 min), which was implemented in hot air ovens arranged in a cascade configuration. Density measurements and optical microscopy verified the success of the foaming. The density of the vulcanised mixtures was the following: REF 1.031 ± 0.001, D 1.161 ± 0.013, D_Fo 0.181 ± 0.032 g/cm^3^. The overall porosity of produced foam is 84.4 ± 2.7%.

**Table 5:** Recipes of the Rubber Compounds for Foaming

material	NBR	PEG	mCF	STA	ZnO	Sul	MBTS	ADCA
D_Fo (phr)	100	4	20	1	5	1.5	2.5	20

The optical micrograph (Figure 12) shows a porous NBR matrix with homogeneous cell distribution in the order of a few hundred micrometres. Milled carbon fibers appear as short, dark filaments embedded in and visible across the rubber matrix. Pores and dispersed fibers suggest an elastomer composite structure with a reduced density due to the foam-like morphology. It may have enhanced reinforcement from the carbon fibers.

**Figure 12:**
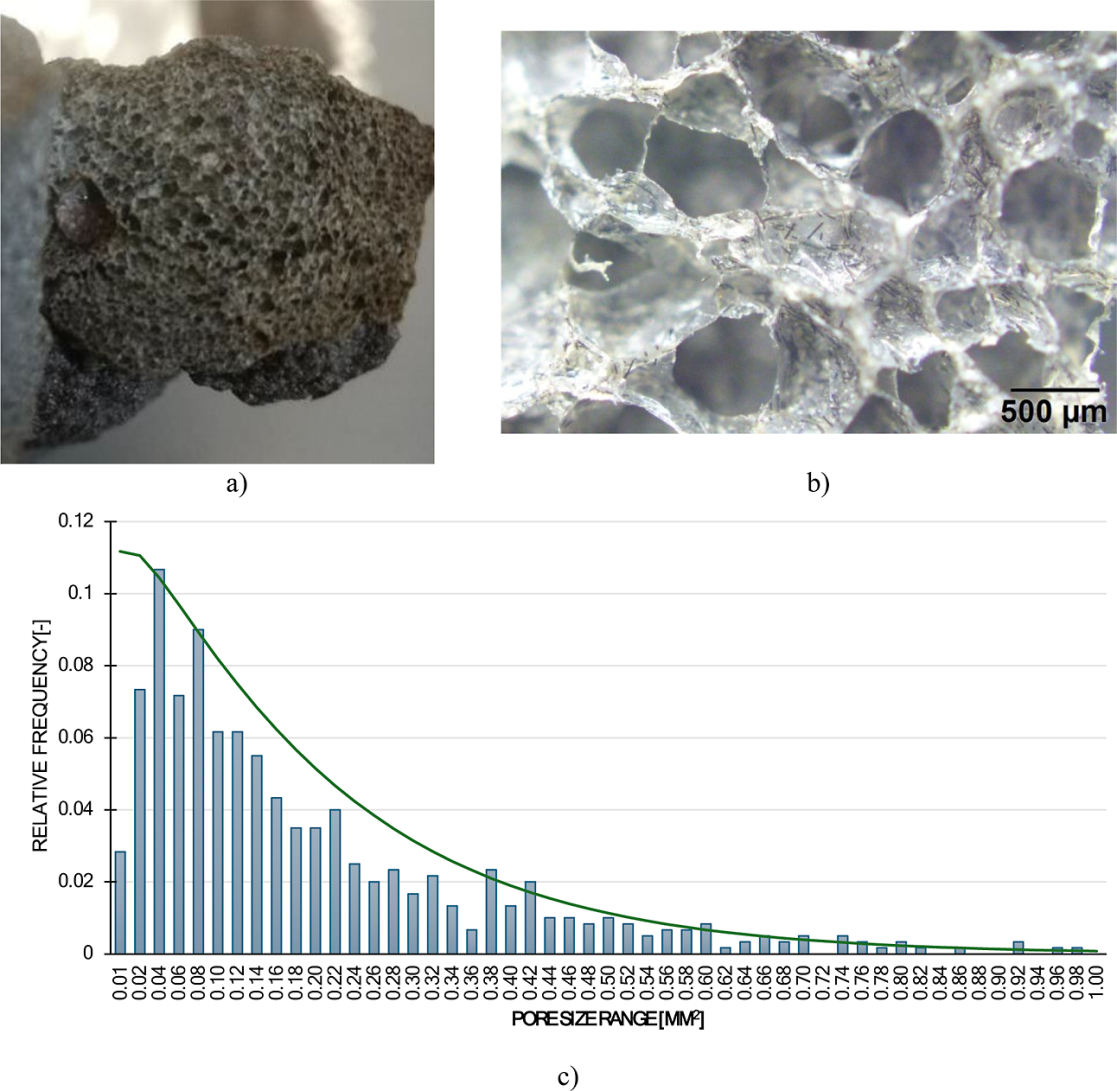
Foamed rubber-based sample containing recycled carbon fibers (a) appearance of rubber foam, (b) microgram of foamed rubber, (c) results of pore size measurements.

## 4 Conclusions

This study aimed to investigate the effects of incorporating recycled milled carbon fiber (rCF) into nitrile butadiene rubber (NBR) composites. The focus was on mechanical performance, curing behavior, wear resistance, and microstructural characteristics. The study’s results demonstrate that rCF retains sufficient structural and chemical integrity even after undergoing mechanical processing to serve as a viable reinforcement in elastomeric systems. The analysis of fiber length distribution revealed that the majority of recycled fibers fall within the 60–74 μm range, with an average length of 70.05 ± 1.12 μm. This size is considered appropriate for enhancing mechanical interlocking within the rubber matrix. FTIR analysis further confirmed that the chemical composition of the recycled fibers remained largely unchanged compared to their virgin counterparts, indicating that the recycling process does not lead to substantial degradation or contamination.

From a curing aspect, rheometric analysis demonstrated that increasing the rCF content significantly improves curing kinetics. This is evidenced by the reduction in characteristic cure times (TC10, TC30, and TC90) and the increase in maximum torque, especially for high rCF content samples. The findings of this study suggest that rCF enhances heat transfer during vulcanization, thereby accelerating cross-linking reactions and enabling more efficient processing.

The incorporation of rCF resulted in a consistent increase in Shore A hardness, which is attributed to the restriction of polymer chain mobility and the formation of a more rigid, cross-linked network. Furthermore, an analysis of tear strength demonstrated a significant positive correlation with rCF content, reaching almost double the resistance of the reference material without reinforcement at the maximum filler loading. These improvements highlight the reinforcing capability of rCF and the ability to distribute and dissipate stresses more effectively within the matrix. However, the tensile strength results revealed a more complex relationship. While moderate rCF contents (10–30 phr) were found to have tensile properties comparable to the reference, further increases resulted in a reduction, likely due to insufficient fiber dispersion, agglomeration, and increased stress concentrations. This finding was verified by SEM imaging, which demonstrated that at elevated rCF loadings, fibers exhibited an aggregation tendency, thereby reducing the uniformity and efficiency of load transfer. The observed cavities in the fracture surfaces provide further indication of partial fiber pull-out, suggesting potential for enhancement of fiber–matrix adhesion. It is interesting to note that the elongation at break remained relatively constant across the range of formulations examined, despite the increase in fiber content. This finding suggests that the rubber phase retains its ductile characteristics, negating the supposition that the stiffening effect of rCF would significantly compromise its flexibility. About the phenomenon of abrasion resistance, it was found that a clear optimum was observed at low rCF content (∼5 phr), with performance deteriorating at higher loadings. This phenomenon can be attributed to the compromise between the reinforcement benefits and the increased surface brittleness caused by fiber agglomerates.

The analysis of fiber orientation demonstrated that intermediate filler contents (20–30 phr) achieve the optimal balance between directional alignment and isotropy. It has been demonstrated that elevated degrees of orientation, particularly at higher contents, can result in anisotropic behavior and the manifestation of localized weaknesses. These findings suggest that to achieve consistent mechanical reinforcement, it is necessary to exercise careful control over the amount of rCF, and its orientation and dispersion.

In conclusion, it can be concluded that recycled carbon fiber can be considered an effective and sustainable additive in rubber composites, offering significant improvements in curing behavior, hardness, and tear resistance. However, the benefits of this process are maximized at intermediate loadings (20–30 phr), beyond which diminishing returns or adverse effects may occur. To realize the potential of rCF in such systems, future work should prioritise improvements in fiber dispersion and interfacial adhesion, particularly through surface modification techniques such as silane-based coupling agents.
